# Persistent Asymptomatic Human Infections by Salmonella enterica Serovar Newport in China

**DOI:** 10.1128/mSphere.00163-20

**Published:** 2020-05-27

**Authors:** Narayan Paudyal, Hang Pan, Beibei Wu, Xiao Zhou, Xin Zhou, Wenqing Chai, Qingqing Wu, Shuning Li, Fang Li, Guimin Gu, Haoqiu Wang, Qinghua Hu, Xuebin Xu, Yan Li, Min Yue

**Affiliations:** a CATG Microbiology & Food Safety Laboratory, Institute of Preventive Veterinary Science & Department of Veterinary Medicine, Zhejiang University College of Animal Sciences, Hangzhou, China; b Zhejiang Province Center for Disease Control and Prevention, Hangzhou, China; c Guangxi Center for Disease Prevention and Control, Nanning, China; d Hangzhou Center for Disease Control and Prevention, Hangzhou, China; e Shenzhen Municipal Center for Disease Control and Prevention, Shenzhen, China; f Shanghai Municipal Center for Disease Control and Prevention, Shanghai, China; g Zhejiang Provincial Key Laboratory of Preventive Veterinary Medicine, Hangzhou, China; h Animal Health Research Division, Nepal Agricultural Research Council, Kathmandu, Nepal; Escola Paulista de Medicina/Universidade Federal de São Paulo

**Keywords:** age, *Salmonella* Newport, antimicrobial resistance, human symptoms

## Abstract

Salmonella enterica serovar Newport (*S*. Newport) infections are gradually on the rise in China from the last decade. For humans’ infections, *S*. Newport has been ranked among the top five serovars responsible for persistent infections, globally. A total of 290 *S.* Newport strains with their relevant clinical metadata were analyzed, and the strains were subjected to whole-genome sequence analysis. Among these, 62.4% (*n* = 181) were from diarrheic patients and 28.9% (*n* = 84) were from asymptomatic individuals (including adults and youngsters) while 8.6% (*n* = 25) were from cases of persistent diarrhea in infants (28%, *n* = 7) and toddlers (72%, *n* = 18). The association between the sequence types (STs) and the variations in the clinical presentation was statistically significant (*P = *0.0432), with ST46 causing diarrhea or representing asymptomatic patients and ST31 or ST68 causing persistent diarrhea. Genomic analysis revealed that the highest proportion of the isolates (98.5%, *n* = 279), primarily from patients with or without diarrhea rather than from asymptomatic individuals, carried antimicrobial resistance determinants corresponding to the aminoglycosides and beta-lactams, highlighting the need for cautionary usage of antimicrobials in such patients. These findings also suggest that cases of nontyphoidal *Salmonella* infection with symptoms of acute diarrhea or persistent diarrhea caused by *S*. Newport should be handled with caution, due to the high chance of development of an antimicrobial resistance phenotype that might lead to therapeutic failures. Together, *S*. Newport ST31 and ST46, which have the highest frequency of carriage of multidrug resistance, are potentially responsible for antimicrobial-resistant diarrhea/persistent diarrhea in infants and children, while adult humans are more likely to be (asymptomatic) carriers of the *S*. Newport strains.

**IMPORTANCE** Human infections caused by *Salmonella* Newport generally lead to gastrointestinal diseases. These infections are normally self-limiting; however, in certain cases, broad-spectrum antimicrobials are prescribed for the treatment. The Chinese National Foodborne Disease Surveillance Network has reported a gradual increase in the incidence of multidrug-resistant *S*. Newport infections in humans. After careful evaluation of the dynamic relationship among the clinical findings, the age group, and the genomic sequence data, it was found that young patients represented the major group with persistent diarrhea, whereas adults were either asymptomatic or diarrheic. Furthermore, all these strains contained multiple acquired antimicrobial resistance determinants, which limited the use of antimicrobials for human patients of all age groups. This analysis of the laboratory-confirmed cases, coupled with genetic analysis of the corresponding pathogen, revealed that antimicrobial treatment of persistent infections by *S*. Newport in infants and toddlers, and in asymptomatic or diarrheic adults, may not be successful. If the antimicrobials must be prescribed at all, they must be used with caution because of the presence of multiple acquired antimicrobial resistance determinants in such strains.

## Abstract (precis)

Human infections caused by *Salmonella* Newport generally lead to gastrointestinal diseases. These infections are normally self-limiting; however, in certain cases, broad-spectrum antimicrobials are prescribed for the treatment. The Chinese National Foodborne Disease Surveillance Network has reported a gradual increase in the incidence of multidrug-resistant *S*. Newport infections in humans. After careful evaluation of the dynamic relationship among the clinical findings, the age group, and the genomic sequence data, it was found that young patients represented the major group with persistent diarrhea, whereas adults were either asymptomatic or diarrheic. Furthermore, all these strains contained multiple acquired antimicrobial resistance determinants, which limited the use of antimicrobials for human patients of all age groups. This analysis of the laboratory-confirmed cases, coupled with genetic analysis of the corresponding pathogen, revealed that antimicrobial treatment of persistent infections by *S*. Newport in infants and toddlers, and in asymptomatic or diarrheic adults, may not be successful. If the antimicrobials must be prescribed at all, they must be used with caution because of the presence of multiple acquired antimicrobial resistance determinants in such strains.

## INTRODUCTION

*Salmonella* is one of the leading causes of diarrhea among all age groups, in particular, for children (<5 years of age) in low-income and middle-income countries (1). Typhoidal *Salmonella* infections can be acute or chronic, and the asymptomatic carrier state has also been reported. For example, nontyphoidal *Salmonella* (NTS) infections typically cause acute self-limiting gastroenteritis in humans; in certain cases, however, such infections can cause invasive febrile bacteremia with fatal outcome. In some cases, a successful treatment could result in recovery from the symptoms but without clearance of the pathogens from the infected individual. Asymptomatic or chronic carrier is defined as a persistent infection, representing a phenomenon that is poorly understood. While cases of chronic carriers of typhoidal salmonellosis are reported frequently (2, 3), those for nontyphoidal salmonellosis are scarce and largely underestimated. In China, the incidence of infections of humans by Salmonella enterica serovar Newport has been gradually on the rise (4–7), and such infections are among those commonly reported from multiple different sources (8, 9).

By using *S.* Newport data from the Chinese National Foodborne Disease Surveillance Network (active surveillance network), we too observed an increasing incidence of persistent and asymptomatic infections in human in China. One Chinese study reported that for the years 2005 to 2011, *S*. Newport was among the top 10 serovars responsible for human clinical cases (10). An Israeli study showed that the persistence index of *S*. Newport in human infections was 1.3 and that *S*. Newport was ranked among the top five serovars (11). The human cases of *S*. Newport and its persistence are generally of low concern in Chinese medical practice due to its nonsignificant clinical outcome in most of the cases. The attitude of the Chinese medical practitioners is probably a consequence of the fact that many enteric bacteria manifest similar clinical symptoms and that the usage of broad-spectrum antimicrobials substantially helps to reduce the severity of the symptoms. Here, we analyzed the clinical and genomic data on *S*. Newport isolates and evaluated the bacterial genetic factors associated with clinical presentations.

## RESULTS

The *S*. Newport strains of human origin, along with the relevant metadata collected from various age groups in China over a period of 18 years (1991 to 2018), were analyzed. The stool samples were collected from human subjects presenting themselves at the health care facilities at 13 provinces, while the Shanghai region contributed the largest number of isolates used in our analysis.

**FIG 1:**
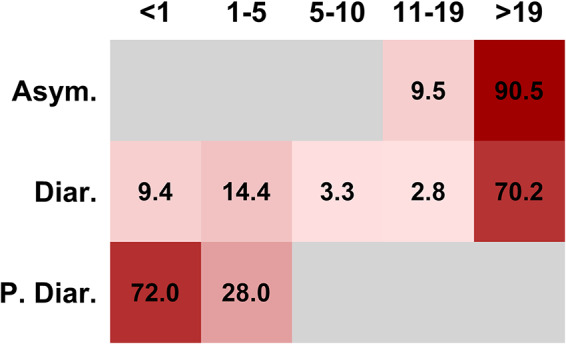
Prevalence by age, symptoms, and clinical findings. Data represent percentages of *S*. Newport (values of individual cells) isolated from different clinical presentations (left side) for various age categories (patient age on top). Blank cells represent the absence of data. The age categories (*x* axis, in years, top) are classified as infant (<1 year of age), toddler (1 to 5 years of age), child (6 to 10 years of age), adolescent (11 to 19 years of age), and adult (>19 years of age). The symptoms (*y* axis) are abbreviated as follows: asymptomatic, Asym.; diarrhea, Diar.; persistent diarrhea, P. Diar.

Among the total 290 strains, the highest proportion, 70% (*n* = 203), were isolated from adults whereas the lowest proportion, 2% (*n* = 6), were from children. Concerning the clinical symptoms, 62.4% (*n* = 181) were from diarrheic subjects whereas 28.9% (*n* = 84) were from asymptomatic individuals and 8.6% (*n* = 25) from individuals with cases of persistent diarrhea. It was a peculiar feature that *S*. Newport was isolated from cases of persistent diarrhea only in infants (28%, *n* = 7) and toddlers (72%, *n* = 18). Adult and other age groups reported only diarrhea or were apparently healthy or asymptomatic (Fig. 1).

The genomic sequence was analyzed for the sequence type (ST) and the acquired antimicrobial resistance genes in 282 strains. Among these 282 strains, 31 different types of known STs along with 6 as-yet-unidentified STs were recorded. The top five STs were ST46 (*n* = 122), ST31 (*n* = 54), ST2364 (*n* = 21), ST166 (*n* = 19), and ST45 (*n* = 17). The six unidentified STs belonged to the strains isolated from stool samples of female adult and children, showing symptoms of diarrhea at Shanghai (2017, 2011, 2010, and 2009, *n* = 1 each), Shenzhen (2009, *n* = 1), and Jiangsu (2010, *n* = 1). ST46 was responsible for nine cases of persistent diarrhea in infants and toddlers and ST31 for seven cases. The analysis also showed that ST46 was isolated mostly from the cases of diarrhea or asymptomatic patients whereas ST31 and ST68 were isolated from the cases of persistent diarrhea. The contribution of ST to the outcome of different clinical presentations was analyzed in two-way analysis of variance (ANOVA). The analysis showed that the ST was a statistically significant (*P = *0.0432) contributor to variation in clinical presentation.

**FIG 2:**
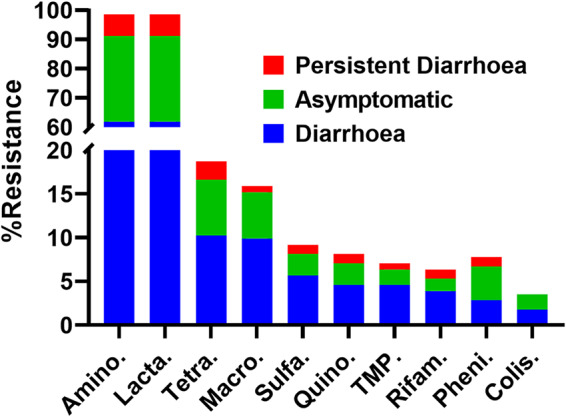
Antimicrobial resistance and clinical findings. Data represent percentages of carriage of acquired resistance genetic determinants (genes) by the *S*. Newport strains isolated from various clinical presentations. The *x* axis gives the classes of antimicrobials as follows: Amino., aminoglycosides; Lacta., beta-lactams; Tetra., tetracyclines; Macro., macrolides; Sulfa., sulfonamides; Quino., quinolones; TMP, trimethoprim; Rifam., rifampin; Pheni., phenicol; Colis., colistin.

The genomic sequence was also scanned for other relevant features such as the number of antimicrobial resistance determinants (ARDs) and the number of mutations. Genomic analysis revealed that the highest proportion of the isolates (98.5%, *n* = 279) carried multiple ARDs corresponding to the aminoglycosides and beta-lactams whereas the lowest proportion of the isolates had carriage of ARDs corresponding to colistin (3.5%, *n* = 10). The genotypic resistance was plotted against the clinical presentation (Fig. 2). This showed that the isolates from patients with symptoms of diarrhea or from asymptomatic patients had the highest rate of carriage of strains with ARDs.

**FIG 3:**
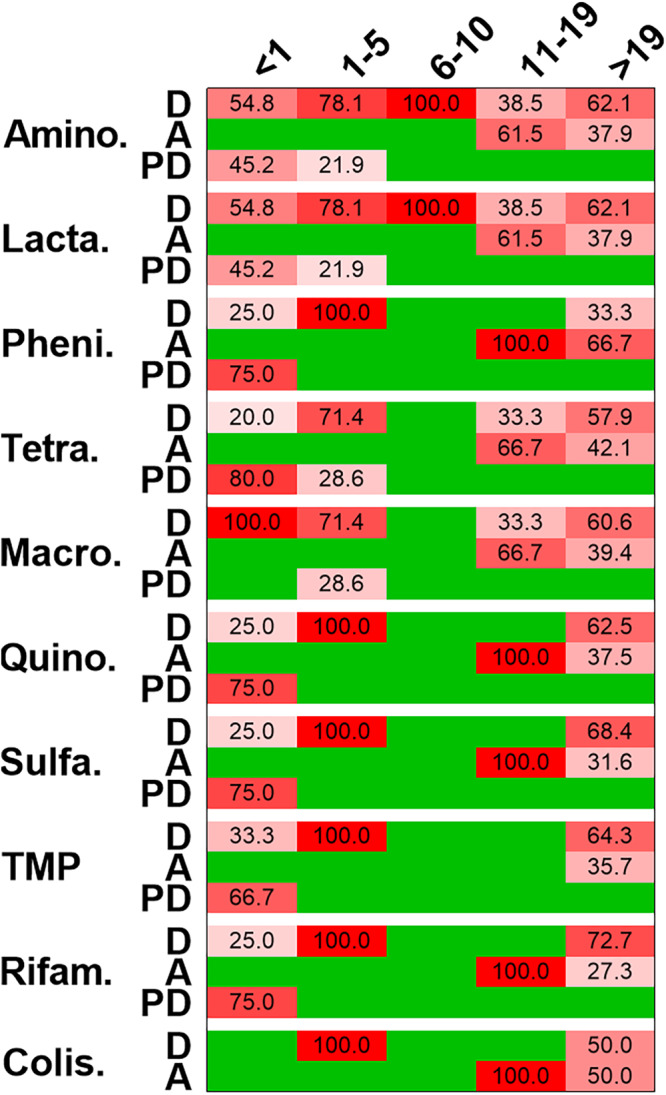
Antimicrobial resistance, age, and clinical findings. The heat map shows the percentages of isolates that were genotypically resistant for the given category of drugs (as described in the Fig. 2 legend) and the clinical symptoms (D, diarrhea; A, asymptomatic; PD, persistent diarrhea) as indicated on the right. The top gives the classifications of age (as described in the Fig. 1 legend). The shades of red show the various percentages of carriage of acquired resistance determinants, while green indicates that the isolates did not contain any such determinants, based on genomic analysis. Values in the cells indicate percentages of carriage of acquired antimicrobial resistance determinants.

Our next aim was to evaluate the relationship between the clinical presentation and the degree of carriage of ARDs in these isolates for various age categories of the patients. The analysis showed that most of the isolates from young patients (<5 years of age) and adults (>19) generally had a higher carriage rate of the ARDs (Fig. 3).

Isolates from the children aged 6 to 10 years generally lacked these ARDs except for those corresponding to the aminoglycosides and the beta-lactams. It was alarming that the range of carriage rates of such ARDs in strains isolated from cases of persistent diarrhea in both the infants and the toddlers was quite high, measuring between 21.9% (aminoglycosides and beta-lactams) and 80% (tetracyclines). Similarly, the carriage rate of such ARDs in asymptomatic adults ranged between 27.3% (rifampin) and 66.7% (phenicols) for various classes of antimicrobial. The details of the presence or absence of the genes conferring resistance to various classes of antimicrobials are provided in Data Set S1 in the supplemental material (see Tab 1). It is notable that, contrary to expectations, there were no mutations in the genes that typically confer resistance to fluoroquinolones. However, mutations in some genes such as Salmonella enterica *gyrA* were found that conferred resistance to fluoroquinolones. D87N was found in four strains isolated from diarrheic patients.

10.1128/mSphere.00163-20.1 DATA SET S1 (Tab 1) Metadata information corresponding to the examined strains used in this study. The different antimicrobial resistance genes (ARGs) predicted by the genomic analysis are also listed. In the table, for each of the ARGs, 0 indicates absence whereas 1 indicates presence (highlighted in yellow). (Tab 2) Prevalence of *bla* genes in the examined isolates. In the table, for each of type of *bla* gene, 0 indicates absence whereas 1 indicates presence (highlighted in yellow). Download Data Set S1, XLSX file, 0.2 MB. Copyright © 2020 Paudyal et al. 2020 Paudyal et al. This content is distributed under the terms of the Creative Commons Attribution 4.0 International license.

All these belonged to the ST31 category and carried 8 to 12 multiple ARGs corresponding to various classes of antimicrobials. Similarly, a *parC* gene that had an S80I mutation conferring resistance to fluoroquinolone and that carried 17 ARGs was seen in only one ST68 isolate. Among the ST11 isolates, Salmonella enterica *gyrA* with a mutation (D87G) conferring resistance to triclosan and carrying seven ARGs was seen only in one strain from a diarrheic patient. Similarly, Salmonella enterica *gyrA* with a mutation conferring resistance to triclosan (S83F) was seen in an array (*n* = 11) of strains isolated from adult diarrheic patients. Among these 11 isolates, 3 belonged to ST31, 6 belonged to ST46, and 2 belonged to ST33. These isolates carried 4 to 11 determinants of resistance to various classes of classes of antimicrobials.

Interestingly, among 285 strains which were detected to be positive for carriage of *bla* genes (Data set S1, Tab 2), we also found that those strains carried *bla*_LEN_ or *bla*_OKP_, both of which are frequently found in *Klebsiella* spp. The results of scanning performed with the newly established toolkit ConFindr for detection of potential contamination showed that, in general, the chance of interspecies sequence contamination was only around approximately 0% to 0.1%, equating to a maximum of only 5 strains at the maximum level.

We calculated the multidrug resistance (MDR) genotypes on the basis of the acquired resistance determinants. In total, 85.2% (*n* = 220 of 258) of the isolates were categorized as MDR. Among these MDR isolates, 50.3% (*n* = 130) of the strains were from diarrheic subjects, 29.8% (*n* = 77) were from asymptomatic subjects, and 5% (*n* = 13) were from the persistent diarrheic subjects. For the various age categories, the MDR strains representing the highest proportion, 40.6% (13 of 32), were isolated from toddlers whereas there were no MDR strains isolated from children’s samples. Among the various STs, the highest MDR frequency was in the ST31 category at 94.4% (*n* = 51 of 94) followed by ST46 at 81.1% (*n* = 99 of 122). Among the remaining STs, the MDR proportion was less than 4%.

## DISCUSSION

A previous study conducted in Guangdong province between 2009 and 2016 reported that children <5 years of age were mostly affected by *Salmonella*; among those children, infants (<1 year) comprised 81.5% (1,084/1,329) of the affected population (12). In another study from Fujian, among the 163 children with *Salmonella* infection, 79 (48.5%) were ≤1 year of age (13). *S*. Newport is one of the most common serotypes isolated from patients with diarrhea (14). Our analysis also shows that infants and toddlers represented the second largest group (23.4%, *n* = 68/290) associated with *S*. Newport after adults (70%, *n* = 203/290). Shanghai was reported to have shown the maximum number of infant *S*. Newport cases (*n* = 28/50) among the various included regions. These data provide additional pieces of evidence for the burden of salmonellosis in infants or younger children caused by *S*. Newport in China.

Here, we show that ST46, ST31, and ST2364 are the top three STs of *S*. Newport reported from human patients with different clinical symptoms. In our earlier study (9), we reported that ST2346, usually including *S*. Newport of freshwater animal origin, is unique to China whereas ST46 and ST31 are primarily associated with reptile-associated cold-blooded animals and seafood-associated animals, respectively. Importantly, this ST46 had been reported from patients in the coastal regions of Shanghai, Fujian, and Guangxi (14).

Evidence hints that many Chinese parents keep turtles as pets. The infants and toddlers usually interact physically and closely with such pets because docile and slow-moving small turtles are seemingly harmless for such interactions. This might explain how the infants and younger children acquired *S*. Newport and thus, in turn, exhibited persistent diarrhea, i.e., due to continuous exposure to the source (the reptilian pet) (15). Though we cannot establish the exact cause-effect relationship, the growing numbers of publications in the literature corroborate our hypothesis (16, 17). Another possible explanation for the longer persistence in children younger than 5 years old might be found in a comparison between the less extensively developed microbiota in children and the more complex microbiota in the adult intestines or even the underdeveloped immune system of such young subjects (18). An earlier report from a study conducted in China also mentioned that *S*. Newport isolates from aquatic products shared high similarity with those from diarrheal patients in several clusters, suggesting a possible association between the isolates derived from these two types of sources (9). Other evidence also shows that seafoods such as fish and turtles, with the latter being generally underestimated players in foodborne transmission, could be among the top sources responsible for human *S.* Newport infections as well as for dissemination of acquired antimicrobial resistance genes such as *mcr* complex genes (4). The actual source (for example, foods, occupational hazards, or environmental contamination, etc.) from which the human subjects might have acquired the pathogens could not be confirmed in the absence of complete historical records in this analysis. Further analyses are being undertaken to evaluate the associations between specific adhesin alleles, host species, and antimicrobial resistance as these have shown potential for diagnosis and epidemiological studies (19).

Carriage of ARDs was found for various members of 10 classes of antimicrobials in these isolates. The carriage rate of aminoglycosides and beta-lactam resistance determinants, as well as determinants of resistance to colistin as reported in an earlier publication (20), is a serious public health concern. Among the antimicrobials, amoxicillin topped the list of the maximally used antimicrobial in human medicine in China (21). This usage might have caused massive selection pressure, thereby resulting in the occurrence of ARDs corresponding to the beta-lactams, and a similar case might be made for the aminoglycosides. In human clinical practice, where enteric pathogens generally manifest with similar symptoms, physicians prescribe the antimicrobials based on their experience rather than relying on time-consuming laboratory findings. Moreover, it is also common for people to visit more than one physician for second opinions, as a consequence of which the subject might be prescribed another type of antimicrobial. This practice rapidly gives rise to MDR strains as seen in these clinical isolates of *S*. Newport. The higher rate of carriage of MDR strains and multiple ADRs in the asymptomatic humans (who are probably carriers) might be a focal point for the spread and distribution of such strains to other human populations where herd immunity might be less than optimal. From the results, it could be seen that the strains from the infants and toddlers with diarrhea or persistent diarrhea had a high rate of MDR carriage. That is an interesting finding because infants are generally less extensively exposed to antimicrobials (clinically) than adults and yet the isolates from them showed a pattern that contrasted with that fact. *Salmonella* pathogens with a broad host range such as *Salmonella* serovar Newport can be transmitted to humans from multiple sources. There have been studies which implicated the allelic variation in multiple adhesins which have some activity in preferential host adaptation, and the use of such variations could have a potential role in source identification (22).

Carriage of *bla*_LEN_ or *bla*_OKP_ is frequently found in *Klebsiella* spp. We also checked sequences for potential interspecies contamination by using the newly established toolkit ConFindr, which has been reported to have the ability to detect cross-species contamination with high sensitivity (23). The results showed that, in general, the chance of interspecies sequence contamination is approximately 0% to 0.1%, equating to only five strains at the maximum level of contamination. Moreover, our sequence coverage was between 78 and 210, further confirming the existence of those genes in *Salmonella* Newport in China. It was well known that class A β-lactamases are usually present only in the chromosome of Klebsiella pneumoniae complex, K. pneumoniae (*bla*_SHV_), K. quasipneumoniae (*bla*_OKP_) and K. variicola (*bla*_LEN_) (24), but recently, similar genes have also been reported in the *Salmonella* Heidelberg serovar of poultry origin (25), and the *bla*_LEN_ gene has also been reported in Staphylococcus epidermis (26). Thus, the presence of these peculiar AMR genes provides novel perspectives on the antimicrobial resistance of *S*. Newport, which warrants further investigations.

This finding suggests that cases of enteric NTS infections caused by *S*. Newport with symptoms of acute diarrhea or persistent diarrhea cannot be treated with antimicrobials, primarily because the bacteria have high carriage of AMR genes that may lead to therapeutic failures. Even for the cases of infant and toddler persistent diarrhea, there were high rates of carriage of AMR genes. It is possible that the adults who were in contact with such infants and toddlers were themselves the carriers, thus contributing to the spread of the AMR pathogen.

On the basis of the available analyses and the evidence obtained here, we can conclude with a high degree of confidence that *S*. Newport ST31 and ST46, which have the highest frequency of carriage of MDR, are potentially responsible for diarrhea/persistent diarrhea in infants and children. The adult human subjects are more likely to be the (asymptomatic) carriers of the *S*. Newport strains. This is a serious public health concern because of the fact that the presence of multiple acquired resistance genes in these isolates potentially jeopardizes the treatment regimen instituted in such cases of persistent diarrhea in infants. Collectively, these findings provide essential knowledge and updated understanding of persistent and asymptomatic infections in patients, which in turn should help efforts to provide better care in primary health care settings, which are able to influence future public health recommendations and health policy planning.

## MATERIALS AND METHODS

We pooled data from the hospital records of human salmonellosis at Shanghai (∼10,000 cases), Shenzhen (∼4,000 cases), and Hangzhou (∼3,000 cases) to analyze the historical strain collection across 13 provinces or province-level municipalities in China. From this library of approximately 17,000 strains, we were able to select only 290 *S.* Newport human strains with their relevant metadata on geographical distribution, age, gender, and major clinical presentation for the descriptive analysis. While the records showed the presence of more than 1,000 strains, there was an absence of relevant metadata for most of those isolates. Those without the related metadata were excluded from further analysis. The oldest strains in the collection dated back to 1991 (*n* = 2), while the recent ones were from 2018 (*n* = 5). The human subjects contributing the fecal samples for bacterial isolation were categorized according to age as infants (<1 year of age), toddlers (1 to 5 years of age), children (6 to 10 years of age), adolescents (11 to 19 years of age), or adults (>19 years of age). The clinical presentations were collated into three main types as individuals with diarrhea (acute, or watery), individuals with persistent diarrhea (lasting >14 days), and apparently healthy (asymptomatic) individuals based on the medical recommendation available along with the relevant metadata. The collected data were curated in Microsoft Excel and analyzed in GraphPad Prism v. 8.0.1 on a Windows platform. A descriptive analysis was performed for age, clinical presentation, and spatiotemporal variation. The interaction between the age and the clinical presentation was analyzed in one-way ANOVA, while the relationship between the top five sequence types and their clinical presentations was analyzed using two-way ANOVA at *P = *0.05. Among these 290 isolates, 282 were subjected to whole-genome sequencing (WGS) on an Illumina HiSeq platform or BGISEQ-500. Multidrug resistance, defined as resistance to three or more classes of antimicrobials, was evaluated using the *in silico* genomic data as described previously (27). Genomic analysis was performed with an in-house script in Python. The WGS data were scanned for potential interspecies contamination by using the newly established toolkit ConFindr (23).

### Data availability.

The summary of the genomic analysis is given in an MS Excel spreadsheet as Data Set S1 in the supplemental material. Other data are available from the corresponding author upon request.

## References

[1] GBD 2016 Diarrhoeal Disease Collaborators. 2018 Estimates of the global, regional, and national morbidity, mortality, and aetiologies of diarrhoea in 195 countries: a systematic analysis for the Global Burden of Disease Study 2016. Lancet Infect Dis 18:1211–1228. doi:10.1016/S1473-3099(18)30362-1. 30243583 PMC6202444

[2] Paudyal N, Yue M 7 1 2019, posting date Antimicrobial resistance in the “dark matter.” Clin Infect Dis doi:10.1093/cid/ciz007. 30615094

[3] Yue M 2016 Bacterial persistent infection at the interface between host and microbiota. Clin Infect Dis 62:1325–1326. doi:10.1093/cid/ciw136. 26980877 PMC4845793

[4] Elbediwi M, Pan H, Biswas S, Li Y, Yue M 2020 Emerging colistin resistance in Salmonella enterica serovar Newport isolates from human infections. Emerg Microbes Infect 9:535–538. doi:10.1080/22221751.2020.1733439. 32122270 PMC7067173

[5] Wang X, Biswas S, Paudyal N, Pan H, Li X, Fang W, Yue M 2019 Antimicrobial resistance in Salmonella typhimurium isolates recovered from the food chain through national antimicrobial resistance monitoring system between 1996 and 2016. Front Microbiol 10:985. doi:10.3389/fmicb.2019.00985. 31134024 PMC6514237

[6] Pan H, Paudyal N, Li X, Fang W, Yue M 2018 Multiple food-animal-borne route in transmission of antimicrobial-resistant Salmonella Newport to humans. Front Microbiol 9:23. doi:10.3389/fmicb.2018.00023. 29410657 PMC5787089

[7] Pan H, Li X, Fang W, Yue M 2018 Analysis of major human and foodborne pathogens and their resistance to antimicrobials in the USA in the past two decades: implications for surveillance and control of antimicrobial resistance in China. J Zhejiang Univ: Agric and Life Sci 44:237–246. doi:10.3785/j.issn.1008-9209.2018.03.124.

[8] Paudyal N, Pan H, Liao X, Zhang X, Li X, Fang W, Yue M 2018 A meta-analysis of major foodborne pathogens in Chinese food commodities between 2006 and 2016. Foodborne Pathog Dis 15:187–197. doi:10.1089/fpd.2017.2417. 29652195

[9] Pan H, Zhou X, Chai W, Paudyal N, Li S, Zhou X, Zhou K, Wu Q, Wu B, Li G, Rajkovic A, Fang W, Rankin SC, Li Y, Xu X, Schifferli DM, Yue M 2019 Diversified sources for human infections by *Salmonella* enterica serovar Newport. Transbound Emerg Dis 66:1044–1048. doi:10.1111/tbed.13099. 30548172 PMC6634944

[10] Wu C, Yan M, Liu L, Lai J, Chan EW, Chen S 2018 Comparative characterization of nontyphoidal Salmonella isolated from humans and food animals in China, 2003–2011. Heliyon 4:e00613. doi:10.1016/j.heliyon.2018.e00613. 29736431 PMC5934692

[11] Marzel A, Desai PT, Goren A, Schorr YI, Nissan I, Porwollik S, Valinsky L, McClelland M, Rahav G, Gal-Mor O 2016 Persistent infections by nontyphoidal Salmonella in humans: epidemiology and genetics. Clin Infect Dis 62:879–886. doi:10.1093/cid/civ1221. 26740515 PMC4787607

[12] Kuang D, Xu X, Meng J, Yang X, Jin H, Shi W, Pan H, Liao M, Su X, Shi X, Zhang J 2015 Antimicrobial susceptibility, virulence gene profiles and molecular subtypes of Salmonella Newport isolated from humans and other sources. Infect Genet Evol 36:294–299. doi:10.1016/j.meegid.2015.10.003. 26440729

[13] Bai L, Guo Y, Lan R, Dong Y, Wang W, Hu Y, Gan X, Yan S, Fu P, Pei X, Xu J, Liu X, Li F 2015 Genotypic characterization of Shiga toxin-producing Escherichia coli O157:H7 isolates in food products from China between 2005 and 2010. Food Control 50:209–214. doi:10.1016/j.foodcont.2014.08.045.

[14] Liang Z, Ke B, Deng X, Liang J, Ran L, Lu L, He D, Huang Q, Ke C, Li Z, Yu H, Klena JD, Wu S 2015 Serotypes, seasonal trends, and antimicrobial resistance of non-typhoidal Salmonella from human patients in Guangdong Province, China, 2009–2012. BMC Infect Dis 15:53. doi:10.1186/s12879-015-0784-4. 25881319 PMC4343067

[15] Harris JR, Neil KP, Behravesh CB, Sotir MJ, Angulo FJ 2010 Recent multistate outbreaks of human salmonella infections acquired from turtles: a continuing public health challenge. Clin Infect Dis 50:554–559. doi:10.1086/649932. 20085463

[16] Hidalgo-Vila J, Díaz-Paniagua C, Pérez-Santigosa N, de Frutos-Escobar C, Herrero-Herrero A 2008 Salmonella in free-living exotic and native turtles and in pet exotic turtles from SW Spain. Res Vet Sci 85:449–452. doi:10.1016/j.rvsc.2008.01.011. 18334260

[17] Marin C, Ingresa-Capaccioni S, González-Bodi S, Marco-Jiménez F, Vega S 2013 Free-living turtles are a reservoir for Salmonella but not for Campylobacter. PLoS One 8:e72350. doi:10.1371/journal.pone.0072350. 23951312 PMC3737154

[18] Gal-Mor O 28 11 2019, posting date Persistent infection and long-term carriage of typhoidal and nontyphoidal Salmonellae. Clin Microbiol Rev doi:10.1128/CMR.00088-18. PMC6302356 30487167

[19] Yue M, Schmieder R, Edwards RA, Rankin SC, Schifferli DM 2012 Microfluidic PCR combined with pyrosequencing for identification of allelic variants with phenotypic associations among targeted Salmonella genes. Appl Environ Microbiol 78:7480–7482. doi:10.1128/AEM.01703-12. 22885744 PMC3457111

[20] Elbediwi M, Li Y, Paudyal N, Pan H, Li X, Xie S, Rajkovic A, Feng Y, Fang W, Rankin SC, Yue M 16 10 2019, posting date Global burden of colistin-resistant bacteria: mobilized colistin resistance genes study (1980–2018). Microorganisms doi:10.3390/microorganisms7100461. PMC6843232 31623244

[21] Zhang QQ, Ying GG, Pan CG, Liu YS, Zhao JL 2015 Comprehensive evaluation of antimicrobials emission and fate in the river basins of China: source analysis, multimedia modeling, and linkage to bacterial resistance. Environ Sci Technol 49:6772–6782. doi:10.1021/acs.est.5b00729. 25961663

[22] De Masi L, Yue M, Hu C, Rakov AV, Rankin SC, Schifferli DM 2017 Cooperation of adhesin alleles in Salmonella-host tropism. mSphere 2:e00066-17. doi:10.1128/mSphere.00066-17. 28289725 PMC5343171

[23] Low AJ, Koziol AG, Manninger PA, Blais B, Carrillo CD 2019 ConFindr: rapid detection of intraspecies and cross-species contamination in bacterial whole-genome sequence data. PeerJ 7:e6995. doi:10.7717/peerj.6995. 31183253 PMC6546082

[24] Rodriguez-Medina N, Barrios-Camacho H, Duran-Bedolla J, Garza-Ramos U 2019 Klebsiella variicola: an emerging pathogen in humans. Emerg Microbes Infect 8:973–988. doi:10.1080/22221751.2019.1634981. 31259664 PMC6609320

[25] Souza AIS, Saraiva MMS, Casas MRT, Oliveira GM, Cardozo MV, Benevides VP, Barbosa FO, Freitas Neto OC, Almeida AM, Berchieri AJ 2020 High occurrence of beta-lactamase-producing Salmonella Heidelberg from poultry origin. PLoS One 15:e0230676. doi:10.1371/journal.pone.0230676. 32231395 PMC7108700

[26] Sheng HH, Qu Y, Wu XM, Dong YY, Zeng XT, Cao H, Huang XW, Yin HQ, Yu YQ, Ni YX, Xiao HS 2008 A new blaLEN-17 gene in a clinical isolate of Staphylococcus epidermidis in Shanghai, China. Chin Med J (Engl) 121:272–275. doi:10.1097/00029330-200802010-00019. 18298924

[27] Hayden HS, Matamouros S, Hager KR, Brittnacher MJ, Rohmer L, Radey MC, Weiss EJ, Kim KB, Jacobs MA, Sims-Day EH, Yue M, Zaidi MB, Schifferli DM, Manning SD, Walson JL, Miller SI 2016 Genomic analysis of Salmonella enterica serovar Typhimurium characterizes strain diversity for recent U.S. salmonellosis cases and identifies mutations linked to loss of fitness under nitrosative and oxidative stress. mBio 7:e00154-16. doi:10.1128/mBio.00154-16. 26956590 PMC4810482

